# Supplementary material for: Splice-Junction-Based Mapping of Alternative Isoforms in the Human Proteome
Source: Cell Rep. Author manuscript; Available in PMC 2020 Jan 15. (PMC6961840; doi:10.1016/j.celrep.2019.11.026)
Supplement: 2 [file NIHMS1546469-supplement-2.zip › DF1/PXD009737/TestisTrypsin/TestisTrypsin_39_CPA5_ELEMVLTATPAQK.pdf]

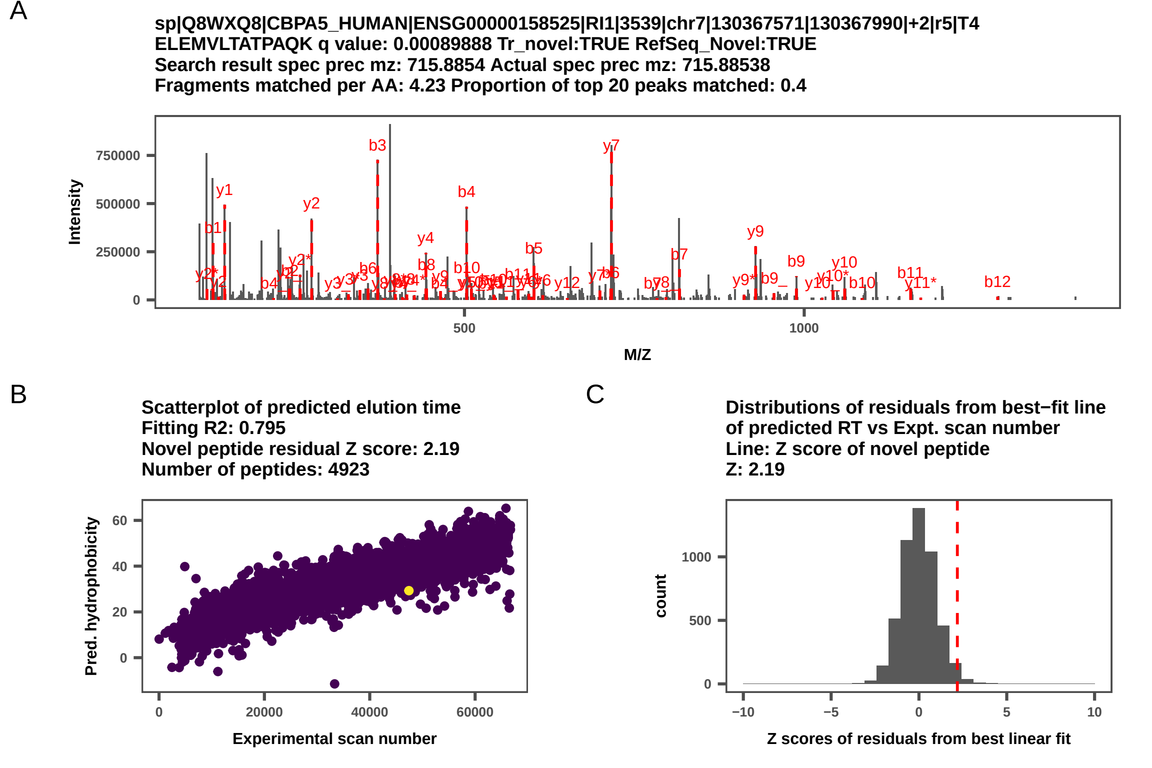
A
sp|Q8WXQ8|CBPA5_HUMAN|ENSG00000158525|RI1|3539|chr7|130367571|130367990|+2|r5|T4
ELEMVLTATPAQK q value: 0.00089888 Tr_novel:TRUE RefSeq_Novel:TRUE
Search result spec prec mz: 715.8854 Actual spec prec mz: 715.88538
Fragments matched per AA: 4.23 Proportion of top 20 peaks matched: 0.4
Intensity
750000
500000
250000
0
500
1000
M/Z
B
Scatterplot of predicted elution time
Fitting R2: 0.795
Novel peptide residual Z score: 2.19
Number of peptides: 4923
Pred. hydrophobicity
60
40
20
0
0
20000
40000
60000
Experimental scan number
C
Distributions of residuals from best−fit line
of predicted RT vs Expt. scan number
Line: Z score of novel peptide
Z: 2.19
count
1000
500
0
−10
−5
0
5
10
Z scores of residuals from best linear fit
